# Flickering in Information Spreading Precedes Critical Transitions in Financial Markets

**DOI:** 10.1038/s41598-019-42223-9

**Published:** 2019-04-05

**Authors:** Hayette Gatfaoui, Philippe de Peretti

**Affiliations:** 1 IÉSEG School of Management (LEM - CNRS 9221), Socle de la Grande Arche, 1 Parvis de La Défense, 92044 Paris, La Défense Cedex France; 2 University Paris 1 Panthéon-Sorbonne, Finance and Modeling Department, Centre d’Economie de la Sorbonne, 106-112 Boulevard de l’Hôpital, 75013 Paris, France

## Abstract

As many complex dynamical systems, financial markets exhibit sudden changes or tipping points that can turn into systemic risk. This paper aims at building and validating a new class of early warning signals of critical transitions. We base our analysis on information spreading patterns in dynamic temporal networks, where nodes are connected by short-term causality. Before a tipping point occurs, we observe flickering in information spreading, as measured by clustering coefficients. Nodes rapidly switch between "being in" and "being out" the information diffusion process. Concurrently, stock markets start to desynchronize. To capture these features, we build two early warning indicators based on the number of regime switches, and on the time between two switches. We divide our data into two sub-samples. Over the first one, using receiver operating curve, we show that we are able to detect a tipping point about one year before it occurs. For instance, our empirical model perfectly predicts the Global Financial Crisis. Over the second sub-sample, used as a robustness check, our two statistical metrics also capture, to a large extent, the 2016 financial turmoil. Our results suggest that our indicators have informational content about a future tipping point, and have therefore strong policy implications.

## Introduction

Over recent years, there has been a growing interest in early warning signals of critical transitions in complex systems, mainly in socio-ecological fields^1–4^. Similarly, financial markets exhibit critical transitions, that is sudden regime shifts that can turn into systemic risk^5^. In particular, systemic risk^6^ refers to connectedness, contagion^7^, cascades, and thus the diffusion of exogenous and/or endogenous shocks within and/or across financial markets^8–12^. Therefore, regulators express a urgent need to anticipate such tipping points, and to compute early warnings of critical transitions, in order to preserve financial stability. Such signals may allow regulators to assess how close to a regime shift a financial system is, and also to evaluate its fragility to an exogenous shock that can drive financial markets to a critical transition.

As regards early warnings in complex financial systems, a recent branch of the literature focuses on the temporal behavior of complex networks to capture changing dynamics. In particular, results suggest that specific changes in the topology of the network convey information about a forthcoming abrupt regime shift. Such an approach relies on the instability in the structure of complex networks^13,14^, and exploits their fast internal changing dynamics^15^, which characterizes highly stochastic systems. For example, in a self-critically organized framework^16,17^, there are strong empirical evidences that a sudden increase in correlations among all components of a financial network precedes a critical transition^14^. Such an increase corresponding to a possible symmetry breaking. Other recent researches focus on a finite set of dyadic and triadic motifs, which characterize the topology of the temporal network^18–20^. They suggest that the time-path of the counts of certain motifs conveys a predictive information about a critical transition. Further, in a temporal and dynamic network^21^, where nodes are connected by short-term causality over each window, total degrees also provide an early warning indicator, increasing long before the occurrence of a critical transition.

In this paper, we relate to the above literature, use a novel empirical approach and introduce new early warning signals. We elaborate on the instability of the topology of a dynamic temporal network, and investigate whether the time-path of information diffusion spreading patterns conveys a relevant information about a forthcoming critical transition^15^. In our dynamic temporal network, over each sliding window, we recover the topology using short-term non-causality tests, two nodes being connected if the null of non-causality is rejected. We use different classes of tests^22,23^ compared to ref.^21^, basing our approach on the cross-correlations of the innovations of time series models. Our procedure also allows to recover the topology while controlling for a possible common driver, thus overcoming the well-known spurious causality problem^24^. Therefore, the topology highlights statistically significant causal relationships between nodes. It is worth emphasizing that other methodologies can be used to represent a simpler sub-graph extracting the most relevant information, and satisfying certain hierarchical structures. These methods include the minimum spanning tree^25,26^ and the planar maximally filtered graph^27^, which provides a larger information set compared to the former method. These methods are typically applied to large datasets and can be tuned through the level of the genus. The major differences with our approach are that we focus on the significance, rather than on the magnitude of the edges and we do not rely on a hierarchical structure to build our network.

Once the topology has been recovered, we focus on information spreading patterns through triangular motifs^28^. We therefore characterize the way nodes communicate with their neighbors in the very short term, and we are able to recover the mapping of information spreading across the dynamic network and over time. The instability of the topology will translate into the variable behavior of nodes, with a fluctuating tendency to be “in” and to be “out” the information diffusion process. We therefore investigate the informational content of the temporal evolution of triangular clustering coefficients about future regimes shifts.

Working at the whole system level, to capture instabilities in information spreading patterns, we build two early warning indicators. Our first indicator is based on the number of switches between clustering and non-clustering phases, some nodes clustering when they enter any motif of information spreading. Our second indicator focuses on the time between two switches^29^.

We highlight two striking features as follows. First, at the vicinity of a critical transition, the stock market being about to switch from one regime to another, both indicators capture flickering in information spreading patterns. We define flickering as sustained periods when nodes rapidly switch between “being in” and “being out” the information diffusion process. Moreover, we statistically gauge the predictive content of our two indicators, and are able to predict a change in a market regime about one year before it occurs. Second, using complementary measures, and specifically before the Global Financial Crisis (GFC), we observe desynchronization^15^ of information spreading patterns across nodes, nodes entering any motif of information spreading at different points in times. The newly defined indicators are thus able to detect regime switches in the stock market. Flickering is informative about a future regime shift while desynchronization is indicative of how close to a tipping point the system is.

In summary, our novel empirical approach allows to build early warning signals that may help regulators going beyond the “too big to fail” or the “too interconnected to fail” policy rule, and moving towards a “too close to a critical transition to fail” rule. In other words, our approach stresses that a forthcoming failure of the whole financial system could rely on the closeness of the system to a critical transition, rather than, or in addition to the failure of a node in an highly interconnected system (i.e. the network’s topology, with cascading effects).

## Methods

To analyze the informational content of the instability in information spreading patterns, we use a multi-step methodology that amounts to (i) Recovering the topology of temporal dynamic networks, where nodes are connected by short-term causality, (ii) Computing clustering measures to analyze information transmission processes, (iii) Building two early warning indicators to track instability in information spreading patterns.

### Causal reconstruction

To test for non-causality, we implement a procedure based on the cross-correlations of the innovations of time series models^22,23^ (See Supplementary Information S1). The procedure has several advantages over more classical tests *a la Granger*^30^. First, being based on innovations of filtered time series, it has a straightforward interpretation in terms of shocks diffusion. Second, it is robust to departures from normality, which are often observed in financial data. Third, the power of the test is not sensitive to additive outliers. Since financial data exhibit heteroskedasticity, we filter the series using generalized autoregressive conditional heteroskedasticity (GARCH) models^31^. Given the stock price index of market *i*: $\{in{d}_{t}^{(i)}\}$ and the associated log-returns ${r}_{t}^{(i)}=\,\mathrm{log}(in{d}_{t}^{(i)}/in{d}_{t-1}^{(i)})$, the GARCH model writes:

1 $${r}_{t}^{(i)}-{\mu }^{(i)}=\sqrt{{h}_{t}^{(i)}}{\varepsilon }_{t}^{(i)}$$

2 $${\theta }_{t}^{(i)}=\sqrt{{h}_{t}^{(i)}}{\varepsilon }_{t}^{(i)}$$

3 $${h}_{t}^{(i)}={\omega }_{{s}_{t}}^{(i)}+{\alpha }_{{s}_{t}}^{(i)}{\theta }_{t-1}^{(i)2}+{\beta }_{{s}_{t}}^{(i)}{h}_{t-1}^{(i)}$$

4 $${\varepsilon }_{t}^{(i)} \sim iid(0,1)$$

where ${h}_{t}^{(i)}$ is the time-varying conditional variance of the log-return ${r}_{t}^{(i)}$; ${\mu }^{(i)}$ is the average log-return; ${\omega }_{{s}_{t}}^{(i)}$, ${\alpha }_{{s}_{t}}^{(i)}$, ${\beta }_{{s}_{t}}^{(i)}$ are state-dependent model parameters; the latent process {*S*_*t*_} is a first-order Markovian process.

We therefore allow parameters in Eq. (3), to switch from one value to another according to two different processes: (i) Recurrent states, i.e., Markov-switching (MS-) GARCH^32^, or (ii) Non-recurrent states, i.e., change-point (CP-) GARCH^33^. Allowing for parameters shifts in the variance equation allows to capture more advanced dynamics and realistic data generating processes. For each time series of the network, we estimate MS-GARCH models with up to three regimes and CP-GARCH models with up to five regimes, and select the model for which the marginal log-likelihood is maximal^34^ (see Supplementary Tables S1 and S2). Total log-returns are then normalized by the square root of their estimated conditional variance (i.e. normalized log-returns).

To build a dynamic temporal network, we use a six-month sliding window. On each window, we first tackle the well-known common driver problem that can induce spurious causality^24^. We orthogonalize the normalized log-return series pairwise with the normalized total log-return of the common driver. The orthogonalization procedure accounts for both the whole causal structure (up to 7 lags) and the instantaneous correlations^35^. Then, considering the normalized and orthogonalized log-returns, we implement non-causality tests based on their cross-correlations^23^, with a lag of plus/minus one day. We then build the asymmetric binary adjacency matrix *A* = {*a*_*ij*_} whose element equals 1 if the null of non-causality from node *i* to node *j* is rejected at the 5% threshold, and 0 otherwise.

### Clustering measures and patterns of information transmission

**Figure 1:**
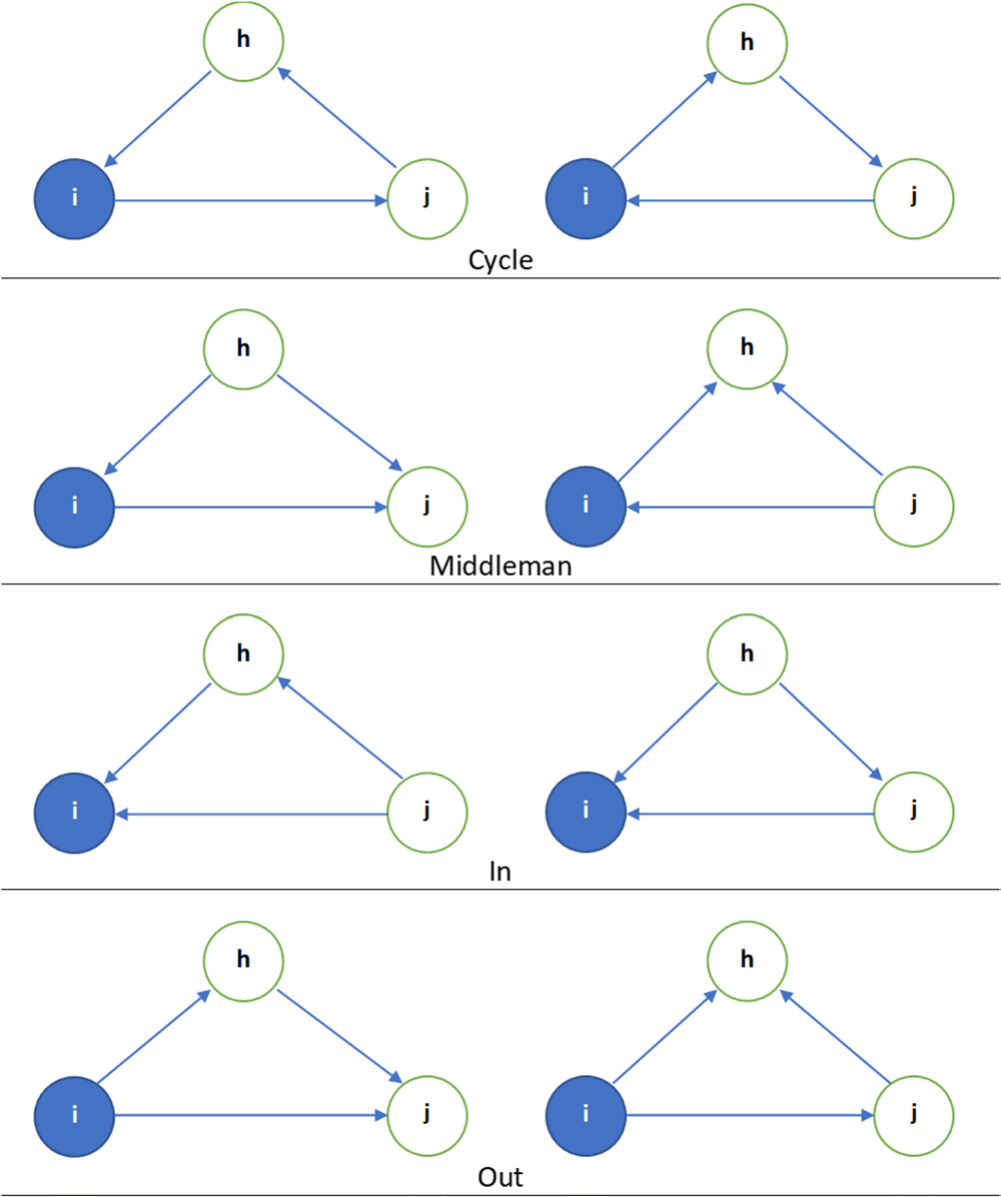
The four triangular motifs^28^. Total clustering is the sum of all possible triangular patterns. The motifs illustrate the ways three nodes can communicate within a network. Several motifs can apply to a given set of nodes at the same time.

Using the asymmetric adjacency matrices, we compute triangular clusters or motifs of information transmission patterns^28^ (See Supplementary Table S3 for computations) over each window, and analyze their evolution over time. As a result, we characterize the way nodes communicate with their neighbors in the very short term. We are then able to recover the mapping of information spreading across the network, and over time (i.e. information spreading patterns in a dynamic network). Figure 1 presents the four types of information transmission patterns. For example, motifs “Out” and “Cycle” correspond respectively to spillover (contagion) and feedback effects. In this paper, we use total clustering which is the aggregation of the eight possible patterns of shocks transmission, as presented in Fig. 1.

### Early warning indicators

For each node *i* and over each window, we compute the total clustering coefficient. To analyze the informational content of its instability over time, we build two indicators. The first one is based on the number of regime switches, and the second one on the time between two switches, when regimes consist of clustering and non-clustering phases. Let’s define ${\{{c}_{t}^{(i)}\}}_{t=1}^{T}$ as the individual total clustering coefficient for node *i*, ${\{{c}_{t}\}}_{t=1}^{T}$ as the average clustering coefficient among all stock exchanges at time *t*, and ${\{{a}_{t}\}}_{t=1}^{T}$ as *a*_*t*_ = 1 if *c*_*t*_ > 0 (clustering phase) and *a*_*t*_ = 0 otherwise (non-clustering phase). When *c*_*t*_ > 0, at least three nodes of the network interact. We define ${\{{d}_{t}\}}_{t=2}^{T}$ as ${d}_{t}=|{\rm{\Delta }}{a}_{t}|=|{a}_{t}-{a}_{t-1}|$, so that *d*_*t*_ is 1 when there is a regime switch, and 0 otherwise. Our first indicator writes:

5 $${S}_{t}^{1}=\lambda {d}_{t}+(1-\lambda ){S}_{t-1}^{1}$$

with *λ* = 0.01 and ${S}_{1}^{1}=\bar{d}$, where $\bar{d}$, is the average value of the indicator of regime switches ${\{{d}_{t}\}}_{t=2}^{T}$.

Equation (5) is an exponentially weighted moving average^36^ (EWMA), computed here over a binary set. This indicator tracks the occurrence of new regime switches, while also accounting for past regime switches. A sustained accumulation of regime switches leads to an increase in the indicator.

Now, let’s define the set $D=\{{t}_{i}|{d}_{{t}_{i}}=1\}$ of switching dates, which are ranked by increasing order. Let ${\tau }_{{t}_{i}}={t}_{i}-{t}_{i-1}$ for ${t}_{i},{t}_{i-1}\in D$ be the inter-events time in days between two consecutive regime switches, that is between two consecutive elements of *D*. Let *q*_0.75_ be the third quartile of the empirical distribution function of inter-events times. Then, as a second indicator, we define a (pseudo) cumulative sum (CUSUM) series as follows:

6 $${S}_{{t}_{i}}^{2}=\,\max (0,{S}_{{t}_{i}-1}^{2}+{q}_{0.75}-{\tau }_{{t}_{i}})$$

with $${S}_{1}^{2}=0$$.

Equation (6) is designed to capture the burstiness of the signal^29^. The shorter the inter-events time becomes, the larger the difference ${q}_{0.75}-{\tau }_{{t}_{i}}$ is, which allows ${S}_{{t}_{i}}^{2}$ to rapidly increase, and hence to capture rapid changes. Conversely, the indicator decreases when the delay between two regime switches becomes large, so that the difference between *q*_0.75_ and ${\tau }_{{t}_{i}}$ becomes negative. In extreme cases, such indicator falls to zero.

In the sequel, by analogy to ecological systems where a system can oscillate between two basins of attraction before a transition occurs, for the whole system, we define flickering by rapid and sustained changes between the two values taken by *d*_*t*_, namely a rapid alternation of 0 and 1 values. At last, analyzing clustering as a signal emitted or received, two nodes *i* and *j* are synchronized if they both enter total clustering over the same time window (${c}_{t}^{(i)} > 0$ and ${c}_{t}^{(j)} > 0$), being in the same cluster or not. These two nodes are said to be de-synchronized if only one node enters total clustering (${c}_{t}^{(i)} > 0$ and ${c}_{t}^{(j)}=0$, or ${c}_{t}^{(i)}=0$ and ${c}_{t}^{(j)} > 0$).

## Results

We implement our methodology on a financial system made of the ten major European stock exchanges. We use total log-returns (indexes between parentheses), namely Belgium (BEL20), France (CAC40), Germany (DAX), Greece (ASE), Italy (FTSEMIB), Ireland (ISEQ), Netherlands (AEX), Portugal (PSI) Spain (IBEX), and United-Kingdom (UKX). Total returns of the United-States (SPX) are used as a proxy for the common driver. All series are downloaded from Bloomberg, on a daily basis from January 1998 to January 2018. We then split our sample into two sub-samples, from January 1998 to May 2014, and from May 2014 to January 2018. The first sub-sample covers four phases of the financial market, the Dot.Com bubble (07 Jan 98–09 Oct 02), the Pre-crisis (10 Oct 02–02 Jul 07), the Global Financial Crisis (03 Jul 07–01 May 09) and the Post-crisis (02 May 09–20 May 14)^37^. The second sub-sample is used as a robustness check over a shorter time window, which is characterized by the 2016 financial turmoil. Over this period, market regimes are estimated using structural break tests in trends^38^, the market undergoing downward trends, sideways and upward trends.

### Main analysis, January 1998 to May 2014

**Figure 2:**
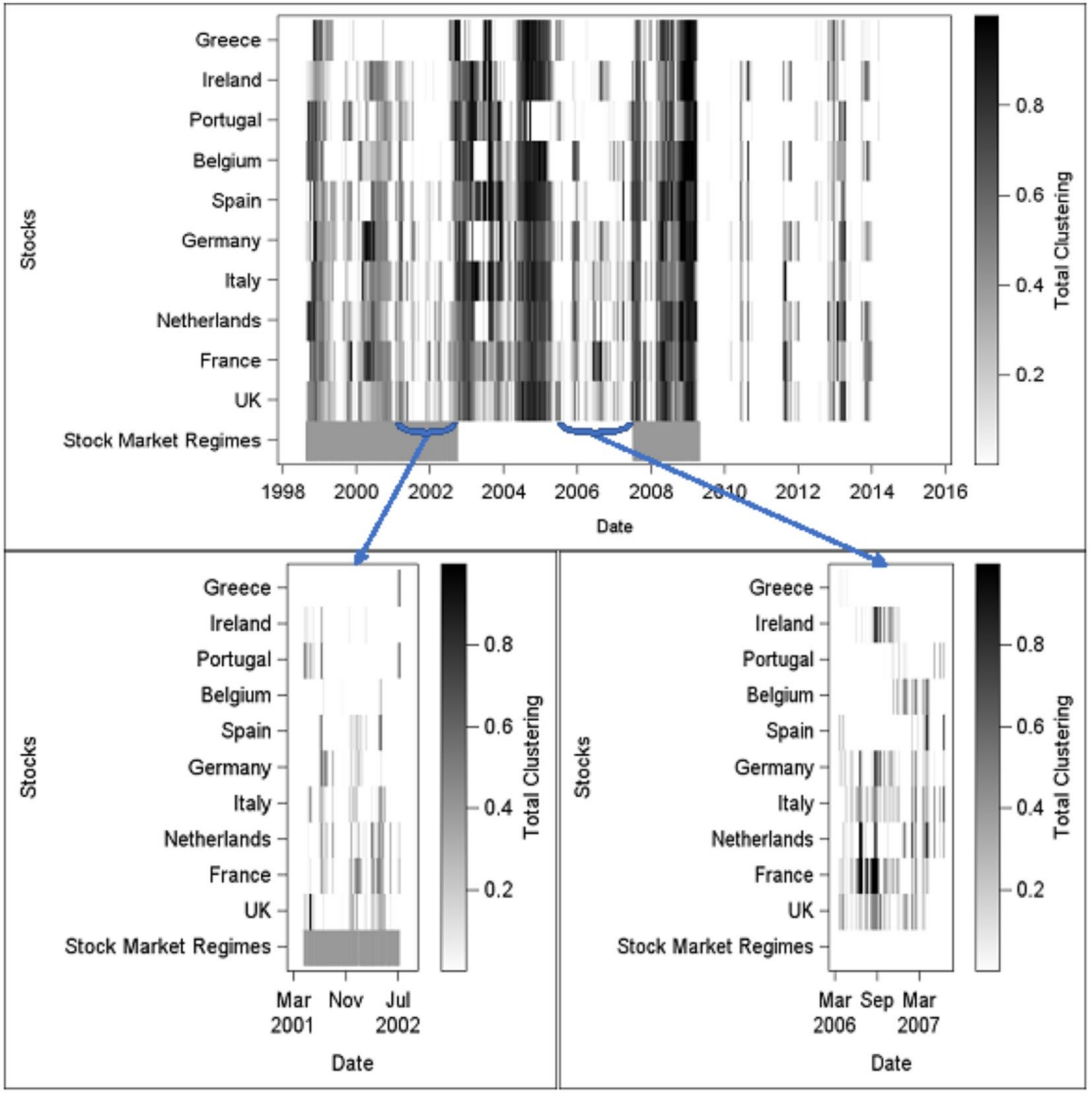
Heatmap of total clustering coefficients (January 1998 to May 2014). Coefficients are scaled between 0 and 1, and computed on a six-month rolling window. Highlighted periods (lower panels) exhibit flickering and desynchronization patterns. “Stock Market Regimes” refers to the four market regimes, i.e. the Dot.com bubble (07 Jan 98–09 Oct 02), the Pre-crisis period (10 Oct 02–02 Jul 07), the Global Financial Crisis (03 Jul 07–01 May 09), and the Post-crisis period (02 May 09–20 May 14).

Figure 2 displays the heatmap of total clustering coefficients, based on a six-month rolling window over the period spanning from January 1998 to May 2014. At the bottom of the graph, the grey and white bands show the different stock market regimes, i.e. regime 1 (grey): Dot.com bubble (07 Jan 98–09 Oct 02); regime 2 (white): Pre-crisis period (10 Oct 02–02 Jul 07); regime 3 (grey): GFC (03 Jul 07–01 May 09), and regime 4 (white): The Post-crisis period (02 May 09–20 May 14). Grey bands highlight periods of market disturbances (i.e. crisis periods). Figure 2 exhibits several interesting features. Overall, two distinct sub-periods, before and after the GFC, are clearly emphasized. While there are strong interactions between markets up to the GFC, such interactions seem to vanish after the GFC, except when the market is hit by external shocks such as the European sovereign debt crisis. Moreover, as expected, the network appears to be highly unstable in terms of information spreading, exhibiting periods of high clustering (at the beginning of regimes 1, 2 and 3, and the end of regime 3) and low clustering (at the end of regimes 1 and 2, see the two lower panels, and inside regime 3). The two low-clustering periods exhibit flickering in information spreading so that each node displays a rapid alternation between “being in” and “being out” the information diffusion process. These periods do precede critical transitions on the market, such phenomenon being particularly striking before the GFC (second highlighted period in the right lower panel). Furthermore, the nodes which enter an information diffusion pattern, become de-synchronized before the occurrence of a critical transition. Hence, flickering and de-synchronization patterns seem to characterize jointly the network prior to a tipping point. At last, regime 2 is of particular interest since it first begins with the clustering of all nodes, then displays an increase in clustering, and finally exhibits flickering clusters. Such evolution could represent pure internal dynamics. It could also be interpreted as the market being hit by an external shock^15^.

**Figure 3:**
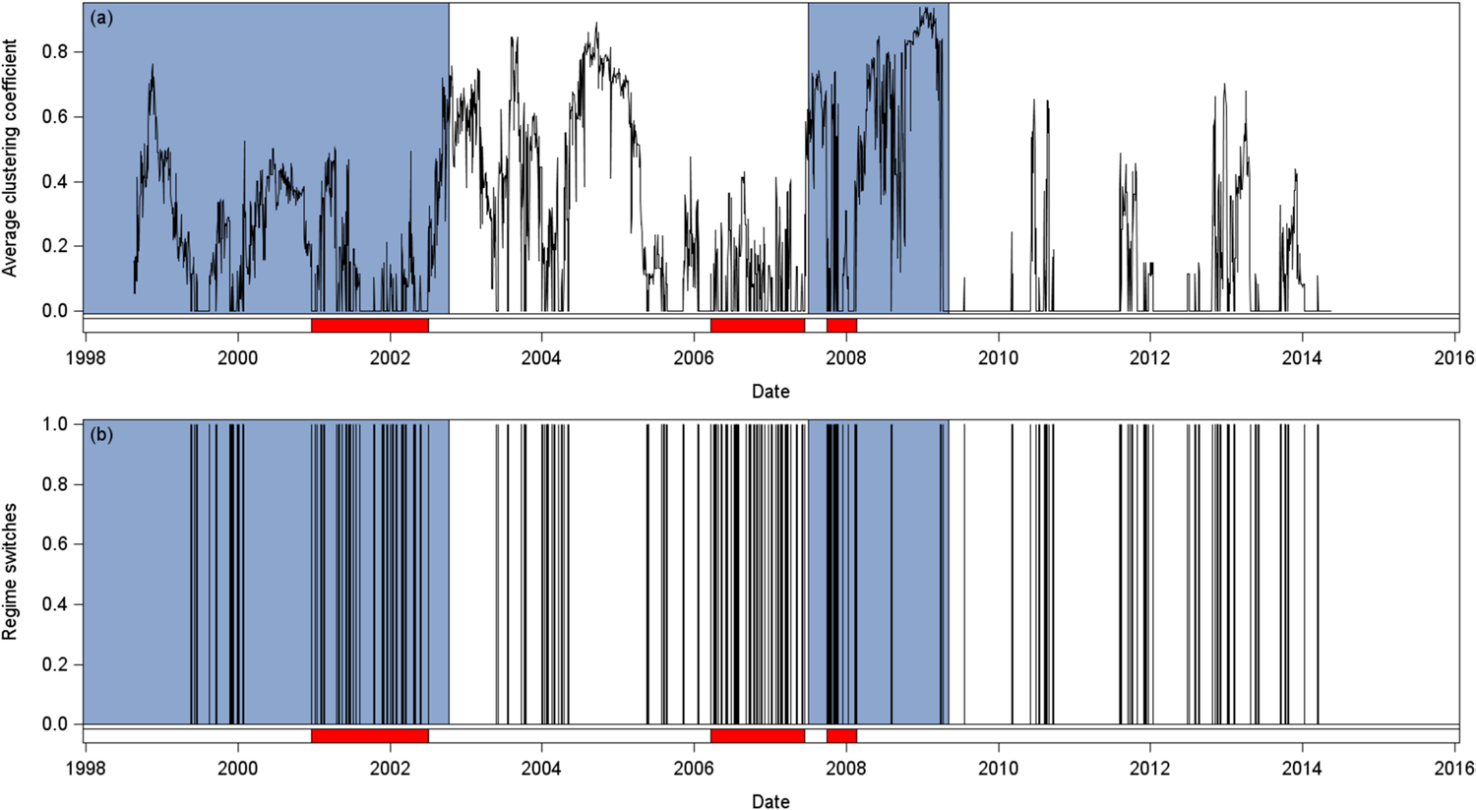
Panel (a) presents the average total clustering coefficient while Panel (b) presents the indicator of regime switches (January 1998 to May 2014). The blue and white areas represent the different market regimes: The Dot.com bubble (07 Jan 98–09 Oct 02), the Pre-crisis period (10 Oct 02–02 Jul 07), the Global Financial Crisis (03 Jul 07–01 May 09), and the Post-crisis period (02 May 09–20 May 14). The blue areas correspond to crisis periods, and the white one to non-crisis periods. The red bands at the bottom of each figure bound flickering periods. At the end of the Dot.com bubble and before the Global Financial Crisis, the signal flickers. High flickering is also observed during the Global Financial Crisis. Overall, the signal is bursty.

Figure 3 shows the average clustering coefficient ${\{{c}_{t}\}}_{t=1}^{T}$ (Fig. 3(a)) and the indicator of regime switches ${\{{d}_{t}\}}_{t=2}^{T}$ (Fig. 3(b)). Red horizontal bars bound flickering periods during each stock market regime (blue and white areas). Before the switch from regime 1 to regime 2, and more obviously, before the GFC, there is a clear evidence of flickering. Furthermore, a high flickering period also takes place during the GFC (i.e. over regime 3).

**Figure 4:**
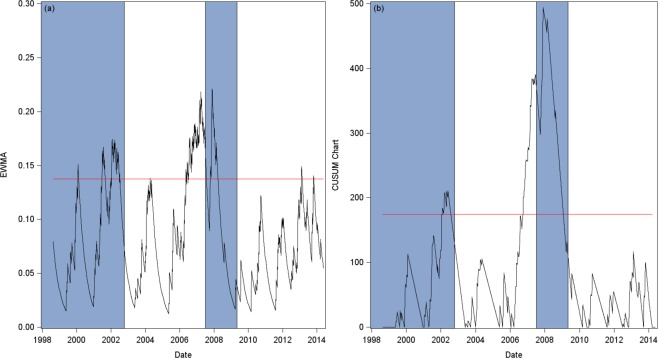
Visual inspection of the two early warnings (January 1998 to May 2014). Panel (a) presents the EWMA of ${\{{d}_{t}\}}_{t=2}^{T}$. Panel (b) plots the CUSUM indicator. On both panels, the blue and white areas highlight the different market regimes: The Dot.com bubble (07Jan98-09Oct02), the Pre-crisis period (10Oct02-02Jul07), the Global Financial Crisis (03 Jul 07–01 May 09), and the Post-crisis period (02 May 09–20 May 14). The red line corresponds to a threshold of three standard deviations.

Figure 4 plots our two early warning indicators together with an upper bound of three standard deviations, which is computed analytically for the EWMA^36^ indicator and by bootstrap for the CUSUM indicator. For the CUSUM indicator, computations yield *q*_0.75_ = 14 days (see Fig. S1), with an extremely left-skewed and fat-tailed distribution of inter-events times (skewness = 5.04, kurtosis = 27.66, median = 5). Blue and white areas still illustrate the different stock market regimes (blue: crisis period, white: non-crisis period). On both charts, threshold exceedances are highly suggestive of a forthcoming critical transition. Such phenomenon is particularly striking for the EWMA indicator as we observe a significant increase before the switch from the Dot.com bubble to the Pre-crisis period, before the GFC with a long period of flickering and at last before the Post-crisis period. Concerning the latter period, flickering is intense and takes place over a short period of time. We note an increased delay between the flickering period and the transition. Recall that the GFC is first triggered by the sub-prime crisis, and then worsens in September 2008 with the collapse of financial institutions such as Lehman brothers. Such contagion effect through bank balance-sheets is of course ignored by our approach. Nevertheless, from a policy point of view, the market is hit after the increase of our indicator, so fragility is high, which translates into downward abrupt changes in prices. This is fully coherent with our model. Overall, the CUSUM chart shows very similar patterns, while also fully capturing the GFC.

#### Predictive analysis

**Figure 5:**
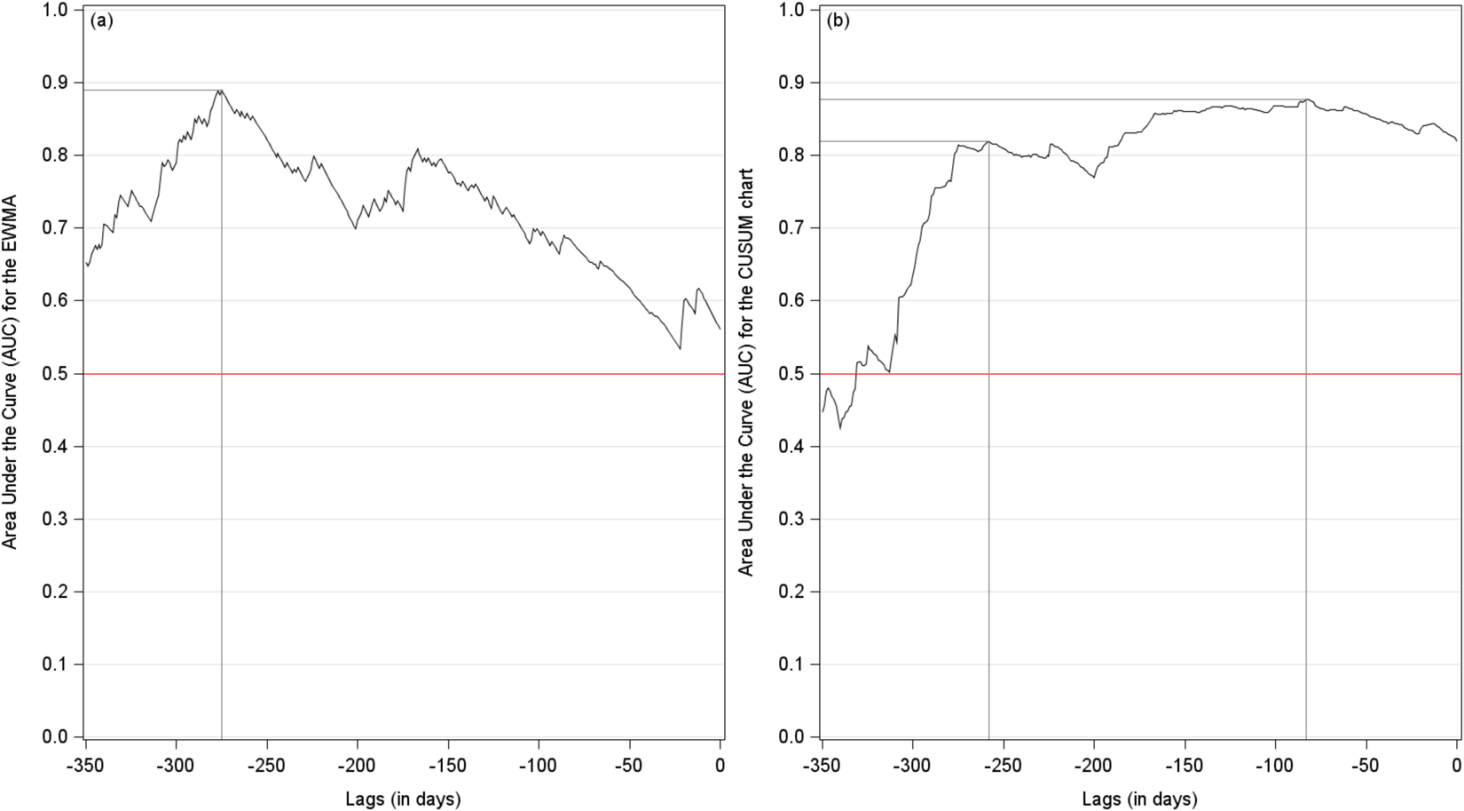
Early warning signals (January 1998 to May 2014). Panels (a) and (b) present the AUCs of the EWMA and CUSUM indicators at different lags, respectively. The AUC of the EWMA indicator reaches a maximum (0.89) at lag −275. The AUC of the CUSUM indicator reaches a first peak at lag −258 (*AUC* = 0.8195), and a second one at lag −83 (*AUC* = 0.8777). Both curves have therefore a high predictive content, about one year before a critical transition occurs.

To confirm our visual inspection, we compute the receiver operating characteristic (ROC) curves and the areas under the curves (AUC) at different lags, ranging from −350 to 0 day(s), for the two indicators above-mentioned. An AUC equals to 0.5 shows no predictive content. Figure 5 reports corresponding results. The AUC of the EWMA indicator reaches a peak at lag −275 days (*AUC* = 0.8897), and then decreases, while approaching 0.5 as the lags tend towards 0. As regards the CUSUM indicator, the behavior of the AUC appears to be more satisfactory, while being much more stable. The AUC reaches a first peak at lag −258 (*AUC* = 0.8195), and a second one at lag −83 (*AUC* = 0.8777). Thus, both indicators may be used as early warning signals since they predict a critical transition about one year before it occurs (a month consists of 22 working days).

**Figure 6:**
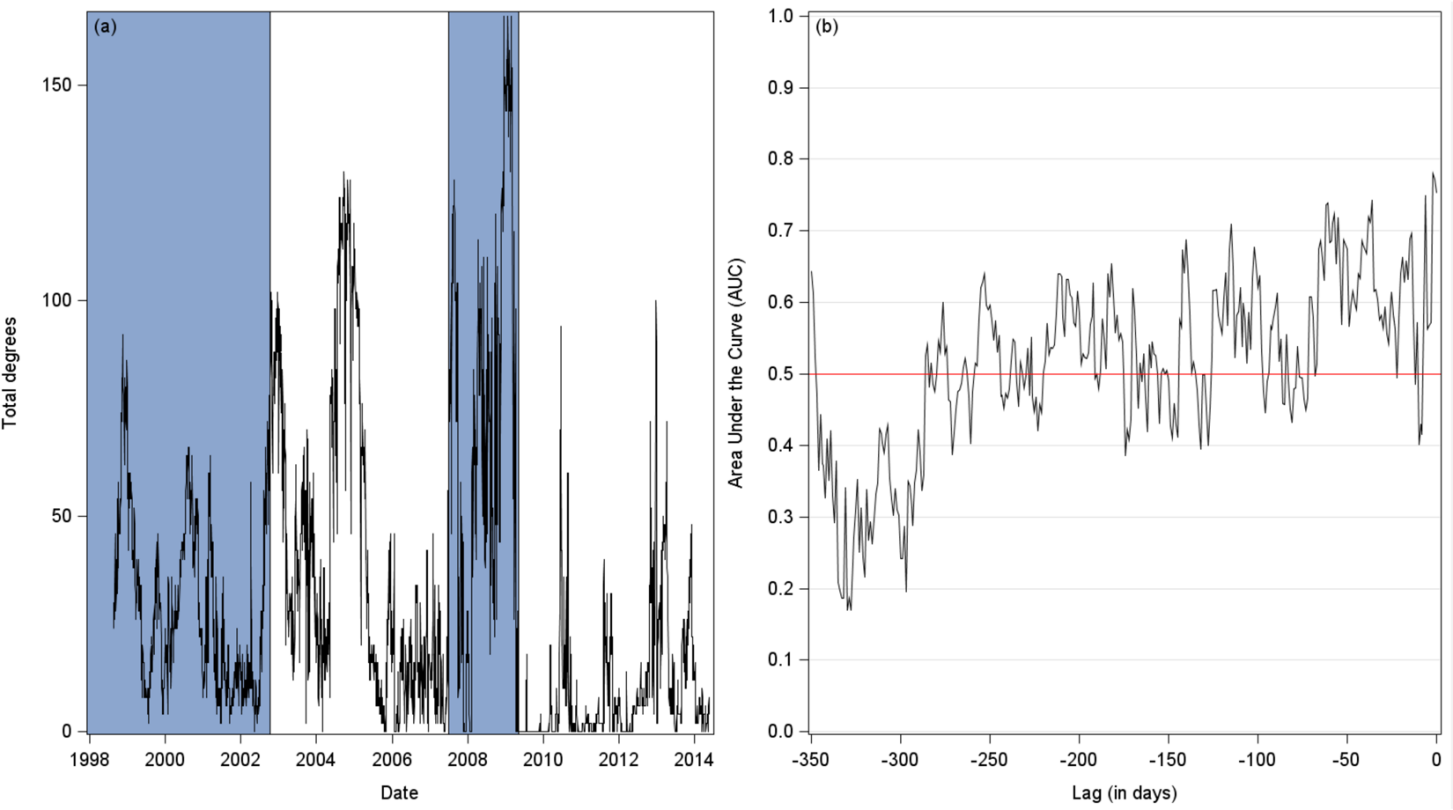
Invalidation of total degrees as an early warning (January 1998 to May 2014). Panel (a) presents the total degrees of the causal network^21^. Panel (b) shows the corresponding AUC. Blue and white areas present the different stock market regimes (blue: crisis, white: Non-crisis). Panel (a) confirms the high instability of the topology, but total degrees have a poor predictive content about a forthcoming critical transition, the different AUCs remaining too close to 0.5, and being unstable.

By way of comparison, the evolution of the total degrees of a short-term causal network^21^ has also been suggested as an early warning. An increase in total degrees being related to a future critical transition. We analyze such an assertion by applying the previous techniques to total degrees. Figure 6 focuses on the total degrees and unveils important features. It confirms the instability of the topology of the temporal causal network, but also shows the erratic behavior of the corresponding AUC. This pattern questions the validity of the total degrees to act as a reliable early warning, at least under our framework. Nevertheless, a period of flickering in total degrees clearly precedes the GFC. Hence, as suggested by our findings, the relevant information in ref.^21^ may not be the increase in total degrees, but rather the instability in total degrees before the GFC.

#### De-synchronization in information spreading

**Figure 7:**
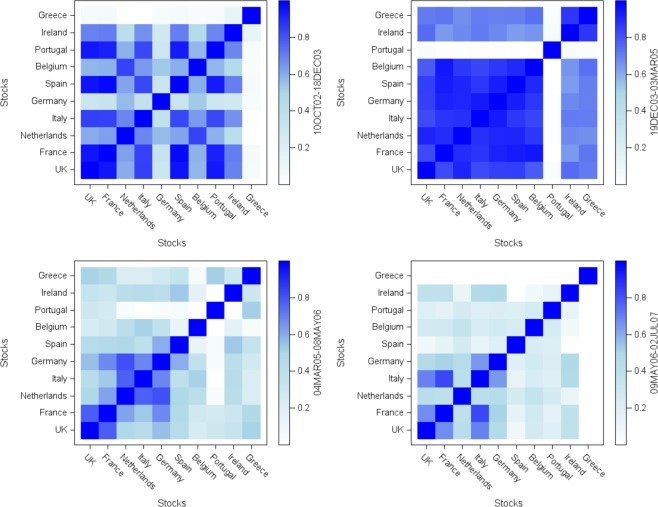
Heatmaps of Jaccard similarity coefficients over four sub-samples of the Pre-crisis period, i.e before the GFC (October 2002 to July 2007). The upper left plot shows high synchronicity, and the upper right plot almost full synchronicity, which is consistent with a possible symmetry breaking^14^. The lower plots show a desynchronization, and highlight a core and a periphery network, the core countries remaining weakly synchronized.

Preceding a critical transition or a tipping point, Figs 2 and 3 show that individual stock exchanges enter a rapid flickering state during the Pre-crisis period. In addition to the rapid flickering, the information diffusion process starts to de-synchronize across nodes, and we observe the asynchronicity of information spreading. Focusing solely on the GFC, we divide the Pre-crisis period into four sub-samples. Over each sub-sample and for each stock exchange, we compute a dummy variable that takes the value of 1 if the corresponding stock index enters a clustering phase, and 0 otherwise. Next, we compute the Jaccard similarity coefficient between pairs of stock exchanges over each sub-sample to capture the transition from synchronicity to asynchronicity. Corresponding results are displayed in Fig. 7. A strong synchronization takes place over the first sub-period, turning to an almost full synchronization over the second period, except for Portugal, which indicates a possible symmetry breaking^14^. Then, the financial system tends to de-synchronize, synchronization becoming weaker over the third sub-sample. Over the fourth and last sub-period, there is a disconnection in information transmission spreading, as shown by the decrease in most pairwise correlations. However, the network still exhibits a weakly synchronous core, comprising Germany, Italy, France, and the UK. Such phenomenon suggests that, just before the Global Financial Crisis, we may have a core network that remains weakly synchronized, and a periphery network, which is fully de-synchronized in terms of information diffusion. This finding is in line with the core/periphery structure of the Eurozone^39^. The core group comprising leading European countries, since France and Germany are the main driving forces of the Eurozone, coupled with the influence of the UK.

### Robustness check, May 2014 to January 2018

As a robustness check, we implement our overall analysis over a second sub-sample, ranging from May 2014 to January 2018. Such recent period covers the 2016 financial turmoil as a strong market disturbance. We use the same panel of countries, apart from Greece, which is excluded, due to quotation disruptions over the recent period. We focus only on the EWMA early warning indicator, and analyze its predictive content about tipping points in both stock index trends and variances. Shifts in trends bound the different market regimes such as downward, sideways and upward trends. To identify the different market regimes, we implement structural break-tests^38^ in trends. We also focus on shifts in variances, gauged using Markov-switching models^34^ (see Supplementary Information). We present corresponding results for three main stock exchanges of the Eurozone: the UK, French and German stock markets.

**Figure 8:**
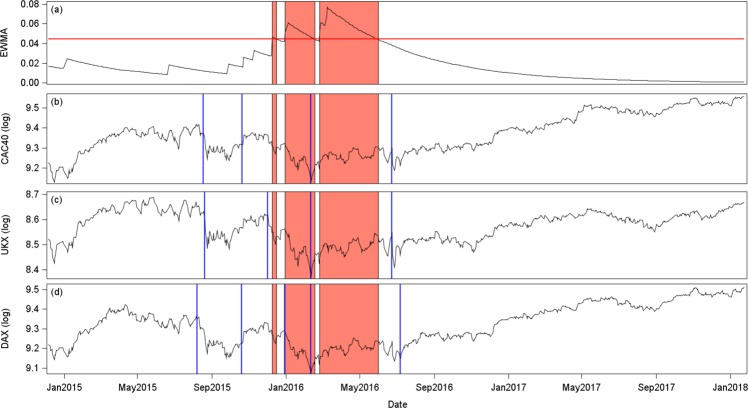
Early warning and stock market trends (May 2014 to January 2018). Panel (a) shows the EWMA early warning indicator, together with a confidence interval of three standard deviations (red line). The orange areas show the periods when the indicator exceeds the confidence interval. Panels (b), (c) and (d) present the logarithm of the total return stock indexes of France, UK and Germany. The vertical blue lines indicate structural breaks, which correspond to tipping points^38^, i.e. changes in trends.

As regards trends and market regimes, Fig. 8 displays the EWMA early warning indicator together with the log-indexes and corresponding break dates (4 breaks for France and UK, and 5 breaks for Germany). The orange areas correspond to periods when the EWMA indicator exceeds the statistical threshold. The two first tipping points of UK and France, and the three first tipping points of Germany are not detected by our early warning indicator. Nevertheless, the two last tipping points are perfectly captured. The first one corresponds to the end of the downward market trend (in mid-February 2016) when the stock market switches from a downward trend to an almost flat one. And, the last one corresponds to a switch from a flat trend to an upward market trend (in late June 2016). Results are particularly striking for France and Germany.

**Figure 9:**
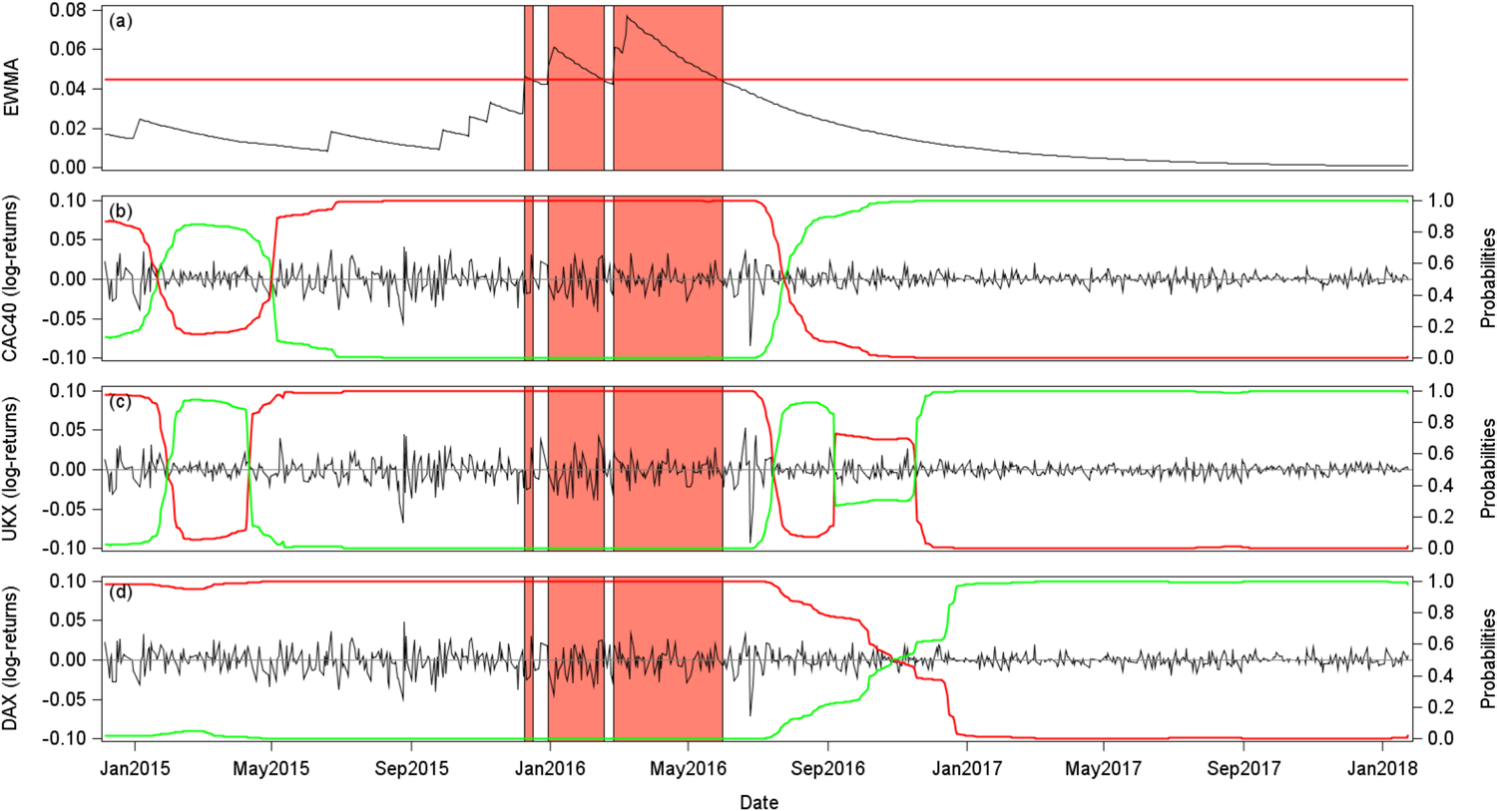
Early warning and stock market volatility (May 2014 to January 2018). Panel (a) shows the EWMA early warning indicator, together with a confidence interval of three standard deviations (red line). The orange areas show the periods when the indicator exceeds the confidence interval. Panels (b), (c) and (d) present the stock index total log-returns together with the probabilities (secondary axis) of being within a state for the conditional variance: Red lines refer to the high volatility regime and green lines refer to the low volatility regime, for France, UK and Germany. When the green (red) line exceeds 0.5, the considered stock market index exhibits a low (high) volatility regime (i.e. low (high) variance state).

Alongside, Fig. 9 focuses on stock index variances. The largest period of flickering (i.e. third orange area) seems to precede a critical transition in variances, at least for France and the UK. It is less obvious for Germany. Therefore, our methodology matches with the stylized fact that an upward trend (last tipping point) in returns is associated with a fall in variance (i.e. stock market recovery, resulting in a growing trend and a reduced risk level).

## Discussion

In this article, we have adopted a novel empirical approach, and introduced several early warning signals to detect critical transitions on the European stock markets. We have therefore considered the multiplicity of market regimes, and we have shown that market shifts were predictable^40^. Our approach aims at analyzing the predictive content of the instability in information spreading patterns with regard to a future tipping point. Using normalized and orthogonalized stock index total log-returns, we have built temporal causal networks. Over each sliding window, we have computed total clustering coefficients for each node of the European stock market. Such clustering coefficients synthesize the information diffusion patterns within the system, and capture complex interactions between nodes and their neighbors, such as spillover or feedback effects. A visual inspection has unveiled the substantial time-variation in the evolution of clustering coefficients. Specifically, the whole system exhibits large synchronized periods of clustering, followed by desynchronized flickering periods of clustering. During these periods, the whole system rapidly switches between clustering and non clustering phases. These switches, referred as flickering periods seem to precede tipping points on the financial market, and the degree of desynchronization informs us about how close to a tipping point the system is. To confirm our visual inspection, we have averaged the individual clustering coefficients, and computed two different indicators. Using ROC curves and AUCs, we have shown that our approach could predict a critical transition about one year before it occurs, both curves reaching an AUC level superior to 0.8. To check the robustness of our approach, we have applied the same methodology to a second sub-sample covering a recent period, which encompasses the significant 2016 financial turmoil. We have shown that we were able to predict two breaks in stock index trends, and a major shift in their corresponding variances.

Our approach, is easy to implement, and can also be used to analyze the fragility of a complex financial system. Our approach could then replace or complete the “too big to fail” or the “too interconnected to fail” policy rule by a “too close to a critical transition to fail” rule. The ability to extract early warnings of forthcoming transitions and crisis should be useful to regulators in order to take preventive measures and mitigate contagion risk. This could be an effective tool in helping regulators carry out their monitoring and supervisory activities.

From a theoretical point of view, our first sub-sample has also been used to test the validity of critical slowing down (CSD) indicators^41^, at the vicinity of a tipping point. In socio-ecological fields, critical slowing down (CSD) indicators have received a great deal of attention^4,42–44^. The CSD indicators are designed to track the lack of resilience of the system resulting in increased first-order auto-correlations and variances. To some extent, CSD indicators may also capture flickering^4,40,45,46^ when the system oscillates between two basins of attraction before a transition occurs. Nevertheless, we were unable to find increasing variances and first-order auto-correlations when approaching the GFC. Other studies have also questioned the validity of CSD indicators when applied to financial systems^47,48^. Such feature may stem from the fact that financial systems are highly stochastic, and critical transitions may be caused by noisy dynamics^15,49,50^. Stochasticity can arise from either an external noise, or random internal dynamics, causing CSD indicators to possibly fail. In such case, flickering in information spreading could reflect a fast noisy dynamics when the system approaches a critical transition.

### Accession codes

Raw data that support the findings of this study are available from Bloomberg but restrictions apply to the availability of these data, which were used under license for the current study, and so are not publicly available. Processed data are however available from the authors upon request, as well as SAS/IML® software codes.

## Supplementary information

Supplementary Information
