# Supplementary material for: Flickering in Information Spreading Precedes Critical Transitions in Financial Markets
Source: Sci Rep. 2019 Apr 5;9:5671. doi: 10.1038/s41598-019-42223-9 (PMC6450864; doi:10.1038/s41598-019-42223-9)
Supplement: Supplementary file 1 — Supplementary Information [file 41598_2019_42223_MOESM1_ESM.pdf]

Supplementary Information

Article in *Scientific Reports*

Flickering In Information Spreading Precedes Critical Transitions in Financial Markets

Hayette Gatfaoui & Philippe de Peretti

Supplementary Discussion S1

Methods

In this section, we detail our statistical procedure to build directed temporal networks.

Data normalization

Financial data exhibit auto-regressive conditional heteroskedasticity[1]. Thus, before implementing causality tests, we need to normalize the series by their conditional variance. The conditional variance is modeled as a generalized auto-regressive conditional heteroskedastic (GARCH) process:

$$r_t^{(i)} - c^{(i)} = \sqrt{h_t^{(i)}}\varepsilon_t^{(i)} \quad (1)$$

$$\theta_t^{(i)} = \sqrt{h_t^{(i)}}\varepsilon_t^{(i)} \quad (2)$$

$$h_t^{(i)} = \omega_{s_t}^{(i)} + \alpha_{s_t}^{(i)}\theta_{t-1}^{(i)2} + \beta_{s_t}^{(i)} h_{t-1}^{(i)} \quad (3)$$

$$\varepsilon_t^{(i)} \sim iid(0,1) \quad (4)$$

where $r^{(i)}$ denotes the total return of stock index i. In the variance equation Eq. (3), the parameters are allowed to switch from one value to another according to two different processes: i) Recurrent states, i.e., Markov-switching (MS-) GARCH[2], or ii) Non-recurrent states, i.e., change-point (CP-) GARCH[3]. Define $S_T = \{s_1, s_2, \ldots, s_T\}'$, where the latent process $\{s_t\}$ is a first-order Markovian process whose transition matrix is defined either by:

$$P_S = \begin{pmatrix} p_{11} & p_{12} & p_{13} & p_{1K} & 1-\sum_{j=1}^K p_{1j} \\ p_{21} & p_{22} & p_{23} & p_{2K} & 1-\sum_{j=1}^K p_{2j} \\ & & & & \\ p_{K1} & p_{K2} & p_{K3} & p_{KK} & 1-\sum_{j=1}^K p_{Kj} \\ p_{K+1,1} & p_{K+1,2} & p_{K+1,3} & p_{K+1,K} & 1-\sum_{j=1}^K p_{K+1j} \end{pmatrix},$$

for a MS-GARCH process, or by:

$$P_C = \begin{pmatrix} p_{11} & 1-p_{11} & 0 & 0 & 0 \\ 0 & p_{22} & 1-p_{22} & 0 & 0 \\ & & & & \\ 0 & 0 & 0 & p_{KK} & 1-p_{KK} \\ 0 & 0 & 0 & 0 & 1 \end{pmatrix}.$$

for a CP-GARCH process where $p_{ij} = P[s_t = j|s_{t-1} = i]$ is the conditional probability to switch from state i on time $t-1$ to state j on time t. We consider MS-GARCH models with up to three regimes, and CP-GARCH models with up to five regimes. Each model is estimated using a Bayesian approach, which combines sequential Monte Carlo (SMC) and Markov chain Monte Carlo (MCMC) methods[4]. After convergence in the Geweke sense[5], we compute the marginal likelihood by bridge sampling (1,000 iterations). The number of particles is set to 150 for change-point models, and to 250 for Markov-switching models. The number of Gibbs iterations is set to 10,000. Finally, we select the model for which the marginal likelihood is maximal. Tables (S1) and (S2) display the marginal likelihoods for the two sub-periods. Over the first sub-period, results support a no-break model for AEX index and a two-break model (i.e., three non-recurrent states) for ASE index. All other stock index

series exhibit a recurrent two-state Markov-switching process (high and low volatility regimes). Over the second sub-period, results are slightly different: AEX, BEL20, CAC40, DAX, UKX and SP500 indexes exhibit a two-regime MS-GARCH process, whereas IBEX is modeled as three-regime MS-GARCH model. ISEQ, PSI and FTSEMIB indexes display a two-regime CP-GARCH representation. Using the above procedure, we build two sets of normalized residuals: $\widehat{\eta} = \{(\widehat{\eta}_t^{(1)}, \widehat{\eta}_t^{(2)}, \ldots, \widehat{\eta}_t^{(10)})' : t \in \mathbb{Z}\} = \{((\widehat{h}_t^{(1)})^{-1/2}\widehat{\theta}_t^{(1)}, (\widehat{h}_t^{(2)})^{-1/2}\widehat{\theta}_t^{(2)}, \ldots, (\widehat{h}_t^{(10)})^{-1/2}\widehat{\theta}_t^{(10)})' : t \in \mathbb{Z}\}$ relating to the ten European stock indexes, and $\widehat{\eta}^{bench} = \{\widehat{\eta}_t^{(11)\prime} : t \in \mathbb{Z}\} = \{((\widehat{h}_t^{(11)})^{-1/2}\widehat{\theta}_t^{(11)})' : t \in \mathbb{Z}\}$ relating to SP500 index.

Causal reconstruction

To recover the topology of a causal network, we use Granger non-causality tests which rely on the cross-correlations of the normalized innovations of filtered time series[6]. Hence, the test has a straightforward interpretation in terms of shocks or information diffusion in the network. Moreover, the statistical procedure is robust to both departure from normality and outliers. Such features describe financial market data.

Generally, let $\widehat{\varepsilon}^{(1)} = \{(\widehat{\varepsilon}_t^{(1)})' : t \in \mathbb{Z}\}$ and $\widehat{\varepsilon}^{(2)} = \{(\widehat{\varepsilon}_t^{(2)})' : t \in \mathbb{Z}\}$ be two sets of innovations of suitable univariate or multivariate time series models, for observed time series $\mathbf{X}^{(1)} = \{(\mathbf{X}_t^{(1)})' : t \in \mathbb{Z}\}$ and $\mathbf{X}^{(2)} = \{(\mathbf{X}_t^{(2)})' : t \in \mathbb{Z}\}$, estimated independently, with values in $\mathbb{R}^{d1}$ and $\mathbb{R}^{d2}$. Define the corresponding covariances and cross-covariances $\mathbf{C}_{\widehat{\varepsilon}}^{(hh)}(0)$ and $\mathbf{C}_{\widehat{\varepsilon}}^{(12)}(k)$ by:

$$\mathbf{C}_{\widehat{\varepsilon}}^{(hh)}(0) = T^{-1}\sum_{t=1}^{T}\widehat{\varepsilon}_t^{(h)}\widehat{\varepsilon}_t^{(h)\prime}, h = 1,2 \tag{5}$$

$$\mathbf{C}_{\widehat{\varepsilon}}^{(12)}(k) = T^{-1}\sum_{t=k+1}^{T}\widehat{\varepsilon}_t^{(1)}\widehat{\varepsilon}_{t-k}^{(2)\prime}, 0 \le k \le T-1, \tag{6}$$

with $\mathbf{C}_{\widehat{\varepsilon}}^{(21)}(k) = \mathbf{C}_{\widehat{\varepsilon}}^{(12)}(-k)'$. The corresponding correlations and cross-correlations at lag k are defined by:

$$\mathbf{R}_{\widehat{\varepsilon}}^{(hh)}(0) = \{\text{diag } \mathbf{C}_{\widehat{\varepsilon}}^{(hh)}(0)\}^{-1/2}\mathbf{C}_{\widehat{\varepsilon}}^{(hh)}(0)\{\text{diag } \mathbf{C}_{\widehat{\varepsilon}}^{(hh)}(0)\}^{-1/2} \tag{7}$$

and

$$\mathbf{R}_{\widehat{\varepsilon}}^{(12)}(k) = \{\text{diag } \mathbf{C}_{\widehat{\varepsilon}}^{(11)}(0)\}^{-1/2}\mathbf{C}_{\widehat{\varepsilon}}^{(12)}(k)\{\text{diag } \mathbf{C}_{\widehat{\varepsilon}}^{(22)}(0)\}^{-1/2} \tag{8}$$

Then, the null hypothesis of no-correlation or independence can be tested using the following portmanteau statistic:

$$\mathbf{Q_M} = T\sum_{k=-M}^{M}\frac{T}{T-|k|}\left(\text{vec } \mathbf{R}_{\widehat{\varepsilon}}^{(12)}(k)\right)'\left(\mathbf{R}_{\widehat{\varepsilon}}^{(22)}(0)\otimes\mathbf{R}_{\widehat{\varepsilon}}^{(11)}(0)\right)^{-1}\left(\text{vec } \mathbf{R}_{\widehat{\varepsilon}}^{(12)}(k)\right) \tag{9}$$

Under the null hypothesis of no-significance of cross-correlations at all leads and lags, Q_M is distributed as a chi-square law with $(2M+1)d_1d_2$ degrees of freedom. Using the above statistic, Granger non-causality tests are easily built by summing over $\{1, M\}$ or $\{-M, -1\}$. For instance, testing for Granger non-causality from $\mathbf{X}^{(2)}$ to $\mathbf{X}^{(1)}$ ($\mathbf{X}^{(2)} \nRightarrow \mathbf{X}^{(1)}$) amounts to computing the following test statistic:

$$Q_M^{+} = T\sum_{k=1}^{M}\frac{T}{T-k}\left(\text{vec } \mathbf{R}_{\widehat{\varepsilon}}^{(12)}(k)\right)'\left(\mathbf{R}_{\widehat{\varepsilon}}^{(22)}(0)\otimes\mathbf{R}_{\widehat{\varepsilon}}^{(11)}(0)\right)^{-1}\left(\text{vec } \mathbf{R}_{\widehat{\varepsilon}}^{(12)}(k)\right) \tag{10}$$

Similarly, to test for non-causality from $\mathbf{X}^{(1)}$ to $\mathbf{X}^{(2)}$ ($\mathbf{X}^{(1)} \nRightarrow \mathbf{X}^{(2)}$), we compute the following statistic:

$$Q_M^{-} = T\sum_{k=-1}^{-M}\frac{T}{T-|k|}\left(\text{vec}\mathbf{R}_{\widehat{\varepsilon}}^{(12)}(k)\right)'\left(\mathbf{R}_{\widehat{\varepsilon}}^{(22)}(0)\otimes\mathbf{R}_{\widehat{\varepsilon}}^{(11)}(0)\right)^{-1}\left(\text{vec}\mathbf{R}_{\widehat{\varepsilon}}^{(12)}(k)\right) \tag{11}$$

Under the null hypothesis, both tests Eq. 10 and Eq. 11 are chi-square distributed with Md_1d_2 degrees of freedom. Hence, in our model, two nodes will be connected if the p-value of Eq. 10 and/or Eq. 11 is less than a given threshold, set here at 5%. All

the above tests are portmanteau tests. For data orthogonalization (see next Section), it is also useful to look at the significance of an individual lead/lag. A natural test statistic is given by:

$$Q(k) = T\frac{T}{T-|k|}\left(\mathrm{vec}\,\mathbf{R}_{\widehat{\varepsilon}}^{(12)}(k)\right)'\left(\mathbf{R}_{\widehat{\varepsilon}}^{(22)}(0)\otimes\mathbf{R}_{\widehat{\varepsilon}}^{(11)}(0)\right)^{-1}\left(\mathrm{vec}\,\mathbf{R}_{\widehat{\varepsilon}}^{(12)}(k)\right) \tag{12}$$

which is also chi-square distributed with $d_1 d_2$ degrees of freedom.

Data orthogonalization

The normalization procedure described above leads to two sets of normalized residuals among which $\widehat{\eta} = \{(\widehat{\eta}_t^{(1)}, \widehat{\eta}_t^{(2)}, \ldots, \widehat{\eta}_t^{(10)})' : t \in \mathbb{Z}\} = \{((\widehat{h}_t^{(1)})^{-1/2}\widehat{\theta}_t^{(1)}, (\widehat{h}_t^{(2)})_t^{-1/2}\widehat{\theta}_t^{(2)}, \ldots, (\widehat{h}_t^{(10)})^{-1/2}\widehat{\theta}_t^{(10)})' : t \in \mathbb{Z}\}$ corresponding to European stock indexes, and $\widehat{\eta}^{bench} = \{\widehat{\eta}_t^{(11)\prime} : t \in \mathbb{Z}\} = \{(\widehat{h}_t^{(11)})^{-1/2}\widehat{\theta}_t^{(11)} : t \in \mathbb{Z}\}$ corresponding to SP500 index. To tackle the omitted variable problem, we consider the SP500 index as a common driver influencing all European stock exchanges, and adopt a two-step procedure[7]. First, for each $k \in \{0, \ldots, M\}$, we compute the test statistics given by Eq. 12 between each component of $\widehat{\eta}$ and $\widehat{\eta}^{bench}$, and keep the significant lags. Then, we regress $\{\widehat{\eta}_t^{(i)\prime} : t \in \mathbb{Z}\}$ on the significant lags of $\{\widehat{\eta}_t^{(bench)\prime} : t \in \mathbb{Z}\}_{t=1}^{T}$, and keep the corresponding residuals $\{\widehat{\varepsilon}_t^{(i)\prime} : t \in \mathbb{Z}\}$. Iterating the procedure for each European stock index, we build a set of ten orthogonalized series $\widehat{\varepsilon} = \{(\widehat{\varepsilon}_t^{(1)}, \widehat{\varepsilon}_t^{(2)}, \ldots, \widehat{\varepsilon}_t^{(10)})' : t \in \mathbb{Z}\}$ with regard to the SP500 index. All tests are implemented on this set of orthogonalized European stock index series. Note that, since the causal structure may evolve over time, the orthogonalization process is performed over each rolling window under consideration.

Clustering measures

A binary network is described by a graph $G = (N, \mathbf{A})$, where N is the number of nodes, here the number of stock indexes, and $\mathbf{A} = [a_{ij}]$ is the $N \times N$ adjacency matrix, where $a_{ij} = 1$ if the null of non-causality hypothesis is rejected at standard threshold, and 0 otherwise. Let $(\mathbf{A})_i$ be the ith row of the matrix, then the in-degree (d_i^{in}), out-degree (d_i^{out}) and total degree (d_i^{tot}) for node i are defined as:

$$d_i^{in} = (\mathbf{A}')_i\mathbf{1} \tag{13}$$
$$d_i^{out} = (\mathbf{A})_i\mathbf{1} \tag{14}$$
$$d_i^{tot} = (\mathbf{A}' + \mathbf{A})_i\mathbf{1} \tag{15}$$

where $\mathbf{1} = (1, 1, \ldots., 1)'$,and the bilateral edges by:

$$d_i^{\leftrightarrow} = \mathbf{A}_{ii}^2 \tag{16}$$

Let T_i^D be the total number of clusters possibly formed by i, the different measures of triangular clustering[8] are given by Table (S3). In this paper, only the total clustering coefficient C_i^D is used.

Supplementary Discussion S2

Results

Supplementary Table S1. GARCH models selection. The Table reports marginal likelihoods for Markov-switching (MS) and change-point (CP) GARCH models computed by bridge sampling, for 10 European stock exchanges, plus the United States. For a given country, the selected model is the one for which the marginal likelihood is maximal. The period spans from August 1998 to May 2014. All series admit a MS-GARCH model with two recurrent regimes, except for ASE index (MS-GARCH model with three regimes, i.e. low, medium and high variance states) and AEX index (no break, i.e. a unique variance regime).

Stock	GARCH	Regimes				
index	Model	1	2	3	4	5
AEX	MS	**-6293.57**	-6294.14	-6296.68		
AEX	CP	-6293.57	-6298.78	-6297.46	-6302.92	-6307.9
ASE	MS	-7484.95	-7440.39	-7462.49		
ASE	CP	-7484.96	-7464.58	**-7433.67**	-7437.97	-7437.66
BEL20	MS	-5855.88	**-5845.54**	-5859.66		
BEL20	CP	-5855.88	-5856.19	-5855.08	-5854.99	-5857.82
CAC40	MS	-6575.57	**-6570.94**	-6576.87		
CAC40	CP	-6575.58	-6579.27	-6579.17	-6576.07	-6582.44
DAX	MS	-6672.93	**-6671.32**	-6680.63		
DAX	CP	-6672.93	-6677.58	-6681.49	-6684.95	-6688.8
FTSEMIB	MS	-6587.34	**-6579.35**	-6587.89		
FTSEMIB	CP	-6587.34	-6586.1	-6579.63	-6579.42	-6589.55
IBEX	MS	-6678.06	**-6669.91**	-6675.54		
IBEX	CP	-6678.15	-6679.26	-6673.07	-6675.38	-6681.4
ISEQ	MS	-6155.85	**-6141.69**	-6157.37		
ISEQ	CP	-6155.83	-6155.25	-6155.85	-6145.67	-6156.4
PSI	MS	-5749.94	**-5728.98**	-5750.49		
PSI	CP	-5749.93	-5747.45	-5732.02	-5736.89	-5731.67
UKX	MS	-5692.76	**-5691.33**	-5698.55		
UKX	CP	-5692.75	-5691.44	-5694.12	-5697.48	-5698.27
SP500	MS	-5775.66	**-5765.51**	-5776.65		
SP500	CP	-5775.66	-5777.91	-5771.92	-5767.62	-5768.15

Supplementary Table S2. GARCH models selection. The Table reports marginal likelihoods for Markov-switching (MS) and change-point (CP) GARCH models computed by bridge sampling, for 10 European stock exchanges, plus the United States. For a given country, the selected model is the one for which the marginal likelihood is maximal. The period spans from May 2014 to January 2016. AEX, BEL20, CAC40, DAX, UKX and SP500 indexes admit a MS-GARCH model with two recurrent regimes, whereas IBEX index results suggest a MS-GARCH model with three regimes. For FTSEMIB, ISEQ and PSI indexes, a CP-GARCH model with a single break (two regimes) is found.

Stock	GARCH	Regimes			
index	Model	1	2	3	4
AEX	MS	-1145.14	**-1138.7**	-1143.83	
AEX	CP	-1145.14	-1142.3	-1141.41	-1144.33
BEL20	MS	-1150.96	**-1145.28**	-1148.68	
BEL20	CP	-1150.96	-1150.09	-1149.11	-1154.72
CAC40	MS	-1295.33	**-1290.98**	-1294.34	
CAC40	CP	-1295.33	-1292.93	-1296.22	-1296.99
DAX	MS	-1319.23	**-1316.76**	-1424.14	
DAX	CP	-1319.23	-1319.98	-1352.30	-1318.13
FTSEMIB	MS	-1562.48	-1558.47	-1561.41	
FTSEMIB	CP	-1562.48	**-1557.27**	-1560.37	-1564.89
IBEX	MS	-1424.61	-1416.24	**-1410.43**	
IBEX	CP	-1424.61	-1417.03	-1419.46	-1427.47
ISEQ	MS	-1255.91	-1254.61	-1256.27	
ISEQ	CP	-1255.91	**-1251.94**	-1254.40	-12561.24
PSI	MS	-1375.91	-1365.34	-1367.96	
PSI	CP	-1375.91	**-1363.22**	-1365.98	-1368.45
UKX	MS	-1246.37	**-1240.64**	-1244.99	
UKX	CP	-1246.37	-1247.19	-1245.03	-1252.01
SP500	MS	-1285.45	**-1166.12**	-1172.38	
SP500	CP	-1285.45	-1176.77	-1178.74	-1173.85

Supplementary Table S3. Triangular patterns and clustering coefficients. T_i^D is the total number of triangular patterns that node i can form.

Patterns	Clustering coefficients for Binary Directed Networks
Cycle	$C_i^{Cyc} = \frac{(A)_{ii}^3}{d_i^{in} d_i^{out} - d_i^{\leftrightarrow}}$
Middleman	$C_i^{Mid} = \frac{(AA'A)_{ii}}{d_i^{in} d_i^{out} - d_i^{\leftrightarrow}}$
In	$C_i^{In} = \frac{(A'A^2)_{ii}}{d_i^{in}(d_i^{in}-1)}$
Out	$C_i^{Out} = \frac{(A^2A')_{ii}}{d_i^{out}(d_i^{out}-1)}$
Total	$C_i^{D} = \frac{(A+A')_{ii}^3}{2T_i^D}$

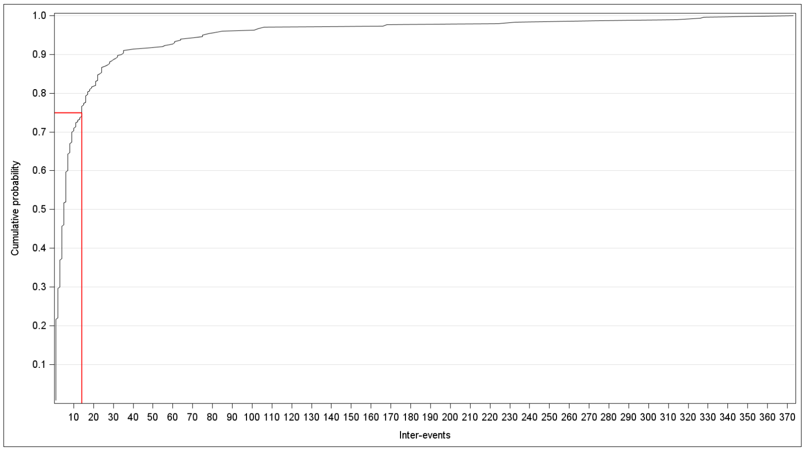

Supplementary Figure S1. Empirical cumulative distribution of inter-events (in days), together with the third quartile value (red lines).

References

1. Bollerslev, T. Generalized autoregressive conditional heteroskedasticity. *J. Econom.* **31**, 307–327, DOI: 10.1016/0304-4076(86)90063-1 (1986).
2. Franq, C. & Zakoian, J. Deriving the autocovariances of powers of markov-switching garch models, with applications to statistical inference. *Comput. Stat. & Data Analysis* **52**, 3027–3046, DOI: 10.1002/jae.2605 (2008).
3. He, Z. & Maheu, J. M. Real time detection of structural breaks in garch models. *Comput. Stat. & Data Analysis* **54**, 2628–2640, DOI: 10.1016/j.csda.2009.09.038 (2010).
4. Bauwens, L., Dufays, L. & Rombouts, J. Marginal likelihood for markov-switching and change-point garch models. *J. Econom.* **178**, 508–522, DOI: 10.2139/ssrn.1965660 (2014).
5. Geweke, J. Interpretation and inference in mixture models: Simple mcmc works. *Comput. Stat. & Data Analysis* **51**, 3529–3550, DOI: 10.1029/2008wr006940 (2007).
6. El Himdi, K. & Roy, R. Tests for non-correlation of two multivariate arma time series. *Can. J. Stat.* **25**, 233–256, DOI: 10.2307/3315734 (1997).
7. Duchesne, P. & Nkwimi, H. On testing for causality in variance between two multivariate time series. *J. Stat. Comput. Simul.* **83**, 2064–2092, DOI: 10.1111/biom.12495 (2013).
8. Fagiolo, G. Clustering in complex directed networks. *Phys. Rev. E* DOI: 10.1093/comnet/cnx057 (2007).
